# Potential Tumor Suppressor NESG1 as an Unfavorable Prognosis Factor in Nasopharyngeal Carcinoma

**DOI:** 10.1371/journal.pone.0027887

**Published:** 2011-11-28

**Authors:** Zhen Liu, Weiren Luo, Ying Zhou, Yan Zhen, Huiling Yang, Xiaoli Yu, Yanfen Ye, Xin Li, Hao Wang, Qinping Jiang, Yajie Zhang, Kaitai Yao, Weiyi Fang

**Affiliations:** 1 Department of Pathology, Basic School of Guangzhou Medical College, Guangzhou, People's Republic of China; 2 Cancer Research Institute, Southern Medical University, Guangzhou, People's Republic of China; 3 Department of Pathology, Guangdong Medical College, Dongguan, People's Republic of China; 4 Third Affiliated Hospital of Guangzhou Medical College, Guangzhou, People's Republic of China; 5 School of Pharmacy, Guangdong Medical College, Dongguan, People's Republic of China; National Taiwan University Hospital, Taiwan

## Abstract

**Background:**

Recently we identified nasopharyngeal epithelium specific protein 1 (NESG1) as a potential tumor suppressor in nasopharyngeal carcinoma (NPC). The purpose of this study is to investigate the involvement of NESG1 in tumor progression and prognosis of human NPC.

**Methodology/Principal Findings:**

NESG1 protein expression in NPC was examined. Survival analysis was performed using Kaplan-Meier method. The effect of NESG1 on cell proliferation, migration, and invasion were also investigated.

**Results:**

NESG1 expression was downregulated in atypical hyperplasia and NPC samples compared to normal and squamous nasopharynx tissues. Reduced protein expression was negatively associated with the status of NPC progression. Patients with lower NESG1 expression had a shorter overall survival and disease-free time than did patients with higher NESG1 expression. Multivariate analysis suggested NESG1 expression as an independent prognostic indicator for NPC patient survival. Proliferation, migration, and invasion ability were significantly increased in cell lines following lentiviral-mediated shRNA suppression of NESG1 expression. Microarray analysis indicated that NESG1 participated in multiple pathways, including MAPK signaling and cell cycle regulation. Finally, DNA methylation microarray examination revealed a lack of hypermethylation at the NESG1 promoter, suggesting other mechanisms are involved in suppressing NESG1 expression in NPC.

**Conclusion:**

Our studies are the first to demonstrate that decreased NESG1 expression is an unfavorable prognostic factor for NPC.

## Introduction

Nasopharyngeal carcinoma (NPC) is a malignant tumor arising from the epithelial cells that line the nasopharynx. Annual NPC incidence rates are less than 1 in 100,000 in most populations, except in southern China, where 20-fold more cases are reported [1]. Synergetic effects of viral infections, genetic alterations, and environmental factors are believed to cause abnormal gene expression which contributes to the development of NPC. Among these changes, the activation of oncogenes and inactivation of tumor suppressor genes may be key steps for initiating tumor formation and development [2]–[7].

We previously compared normal human nasopharynx mucosa and oral cavity mucosa of the soft palate using differential display and identified full-length NESG1 (Accession number: NM_012337.1; Official name:CCDC19) specifically expressed in human nasopharynx and trachea [8]. In a subsequent investigation, we revised the open reading frame (ORF) sequence of NESG1 and updated its version number from NM_012337.1 to NM_012337.2 in the NCBI GeneBank database [9]. NESG1 was found to be specifically expressed in the nasopharynx epithelium and decreased or absent in NPC tissues and cell lines compared to normal tissue. In addition, the levels of NESG1 protein were significantly greater in the lower-grade NPC tissues versus higher-grade NPC. Elevated expression of NESG1 in NPC cells not only significantly decreased cell proliferation and G1-S phase transition, but also markedly inhibited cell migration, invasion, and *in vivo* tumorigenesis. NESG1 also significantly regulated the expression of cell cycle regulators CCNA1 and p21. Our findings provided evidence that NESG1 may act as a tumor suppressor in NPC [9].

In this study, we present further evidence that NESG1 protein is downregulated in human NPC tissues and NPC cells compared to noncancerous nasopharynx tissues. We also show that reduced protein expression of NESG1 is inversely associated with NPC progression and poor prognosis. Downregulation of overexpressed NESG1 in 2F4 NPC cells significantly regained cell proliferation, migration, and invasion. Furthermore, NESG1 knockdown elevated CCNA1 expression and suppressed p21 expression. Gene expression profile analysis indicated that NESG1 participates in multiple pathways, such as MAPK signaling and tight junction formation regulation. Finally, an epigenetic evaluation of the NESG1 promoter revealed a lack of methylation, suggesting involvement of other mechanisms in NESG1 suppression during NPC. Our studies firstly demonstrate that NESG1 as a potential tumor suppressor is an unfavorable prognostic factor for NPC.

## Results

### Downregulated protein expression of NESG1 was associated with NPC progression

**Figure 1:**
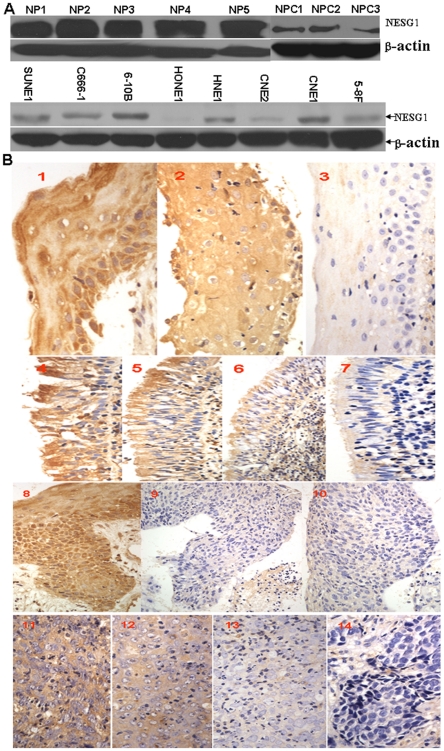
Gradual downregulation of NESG1 protein in normal nasopharynx tissues, atypical hyperplasia, and NPC samples A. Western blot analysis indicated that NESG1 protein was significantly downexpressed in NPC tissues and NPC cells compared to noncancer nasopharynx epithelial tissues. **B.** NESG1 expression was progressively decreased in atypical hyperplasia and NPC samples compared to normal nasopharynx and squamous tissues. **1**) NESG1 expression in squamous epithelium: 1–2: Strong expression, 3: Weak expression; **2**) NESG1 expression in normal epithelium: 4–5: Strong expression, 6: Positive expression, 7: Weak expression; **3**) NESG1 expression in atypical hyperplasia tissue: 8: Strong expression, 9–10: Weak expression; **4**) NESG1 expression in NPC: 11: Strong expression, 12: Positive expression, 13: Weak expression, 14: Negative expression.

We measured the protein expression levels of NESG1 in 8 NPC cell lines, 3 NPC tissues, and 5 noncancerous nasopharynx samples by western blot. Markedly reduced expression of NESG1 was observed in NPC cell lines and tissues compared to noncancerous nasopharynx samples (Figure 1A). Expression levels of NESG1 were then measured in samples of 204 archived paraffin-embedded NPC, 74 normal nasopharynx, 40 squamous epithelium, and 35 atypical hyperplasia using immunohistochemical staining (Figure 1B1–14). NESG1 protein was highly expressed in normal and squamous epithelium samples (*P* = 0.732) compared to atypical hyperplasia and NPC samples (*P*<0.001). Furthermore, NESG1 expression in atypical hyperplasia samples was relatively higher than in NPC samples (*P* = 0.021)(Table 1). Further, we analyzed the relationship between clinicopathologic characteristics and NESG1 expression levels in individuals with NPC (Table 2). We did not find a significant association of NESG1 expression levels with patient's age, sex, smoking status, family tumor history, patients from area, or tumor size (T classification) in 204 NPC cases. However, we observed that the reduced expression level of NESG1 was markedly correlated with lymph node metastasis (N classification) (N0–N1 *vs.* N2–N3) (*P* = 0.005), distant metastasis (N classification)(*P* = 0.024), and clinical stage (I–II *vs.* III–IV) (*P* = 0.003) in NPC patients.

**Table 1:** Gradual downregulation of NESG1 protein in atypical hyperplasia and NPC samples compared to normal nasopharynx tissues.

Group	Protein expression (n)					P Value				
	Total	Negative	Weak	Positive	Strong	S/N/A/C	S/N	A/N	C/N	A/C
S	40	0	3	13	24					
N	74	0	4	29	41	0.008*	0.732#	0.000#	0.000#	0.021#
A	35	0	12	19	4					
C	204	26	87	67	24					

*Kruskal Wallis Test;

#Mann-Whitney U Test; S: Squamous epithelium; N: Normal epithelium; A: Atypical hyperplasia epithelium; C: Cancer; n: Patient case.

**Table 2:** Correlation between the clinicopathologic characteristics and expression of NESG1 protein in NPC.

Characteristics	n	NESG1 (%)		*P*
		High expression	Low expression	
Gender				
Male	142	64 (45.1)	78 (54.9)	0.879
Female	62	27 (43.5)	35 (56.5)	
Age (y)				
≥50	96	42 (43.8)	54 (56.2)	0.888
<50	108	49 (45.4)	59 (54.6)	
Smoking				
Yes	44	19 (43.2)	25 (56.8)	0.865
No	160	72 (45.0)	88 (55.0)	
Family tumor history				
Yes	11	8 (72.7)	3 (27.3)	0.063
No	193	82 (42.5)	111 (57.5)	
Patients from area				
Patients from zhongshan	34	13 (38.2)	21 (61.8)	0.454
Patients from non-zhongshan	170	78 (45.9)	92 (54.1)	
T classification				
T_1_–T_2_	146	70 (47.9)	76 (52.1)	0.117
T_3_–T_4_	57	20 (35.1)	37 (64.9)	
N classification				
N_0_–N_1_	107	58 (54.2)	49 (45.8)	0.005
N_2_–N_3_	97	33 (34.0)	64 (66.0)	
Distant metastasis				
Yes	14	2 (87.5)	12 (12.5)	0.024
No	190	89 (8.30)	101 (91.7)	
TNM Clinical stage				
I∼II	91	37 (40.7)	54 (59.3)	0.003
III∼IV	113	24 (21.2)	89 (78.8)	

### Survival analysis

**Figure 2:**
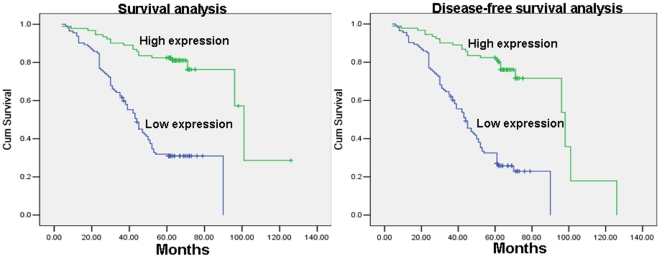
Reduced expression of NESG1 decreases the survival time and disease-free survival time of NPC patients **A.** 1. Lower levels of NESG1 reduced the survival time of NPC patients; 2. Decreased NESG1 expression diminished the disease-free survival time of NPC patients.

To investigate the prognostic value of NESG1 expression for NPC, we assessed the association between protein levels and patients' survival using Kaplan–Meier analysis with the log-rank test. In 204 NPC cases with prognosis information, we observed that the level of NESG1 protein expression was significantly correlated with overall survival, as patients with lower levels of NESG1 expression had poorer survival (Figure 2A1) and shorter disease-free survival duration (Figure 2A2) than those with higher NESG1 expression (*P*<0.001). In addition, age, TNM classification, and clinical stage were also significantly correlated with patients' survival (*P* = 0.034, *P*<0.001, *P* = 0.003, *P*<0.001, and *P*<0.001 respectively). We performed multivariate analysis of the levels of NESG1 protein expression adjusted for age, TNM classification, and clinical stage of NPC patients to determine that NESG1 expression was an independent prognostic factor for NPC (*P*<0.001) (Table 3).

**Table 3:** Summary of univariate and multivariate Cox regression analysis of overall survival duration.

Parameter	Univariate analysis			Multivariate analysis		
	*P*	HR	95%CI	*P*	HR	95%CI
**Gender**						
Male vs. female	0.244	1.313	0.831–2.076			
**Age**						
≥50 vs. <50 years	0.034	1.543	1.033–2.305	0.034	1.554	1.034–2.336
**Family tumor history**						
Yes vs. No	0.083	0.290	0.071–1.178			
**Smoking**						
Yes vs. No	0.282	1.289	0.811–2.047			
**Area**						
Zhongshan vs. Non-Zhongshan	0.161	0.702	0.428–1.151			
**Career**						
Employee of Government Vs. Non-Gonverment	0.842	0.912	0.371–2.247			
**Biotherapy**						
Yes vs. No	0.193	0.393	0.096–1.604			
**T classification**						
T_1_–T_2_ vs. T_3_–T_4_	0.000	2.209	1.462–3.338	0.007	2.004	1.209–3.321
**N classification**						
N_0_–N1 vs. N_2_–N_3_	0.003	1.854	1.236–2.781	0.031	1.807	1.055–3.095
**M classification**						
M_0_ vs. M_1_	0.000	6.051	3.264–11.220	0.000	4.006	2.093–7.664
**Clinical stage**						
I–II vs. III–IV	0.000	2.656	1.566–4.505	0.758	0.887	0.415–1.897
**NESG1 expression**						
High expression **vs.** Low expression	0.000	0.189	0.112–0.317	0.000	0.244	0.143–0.418

### Reduced NESG1 expression increases proliferation of NPC cells *in vitro*

**Figure 3:**
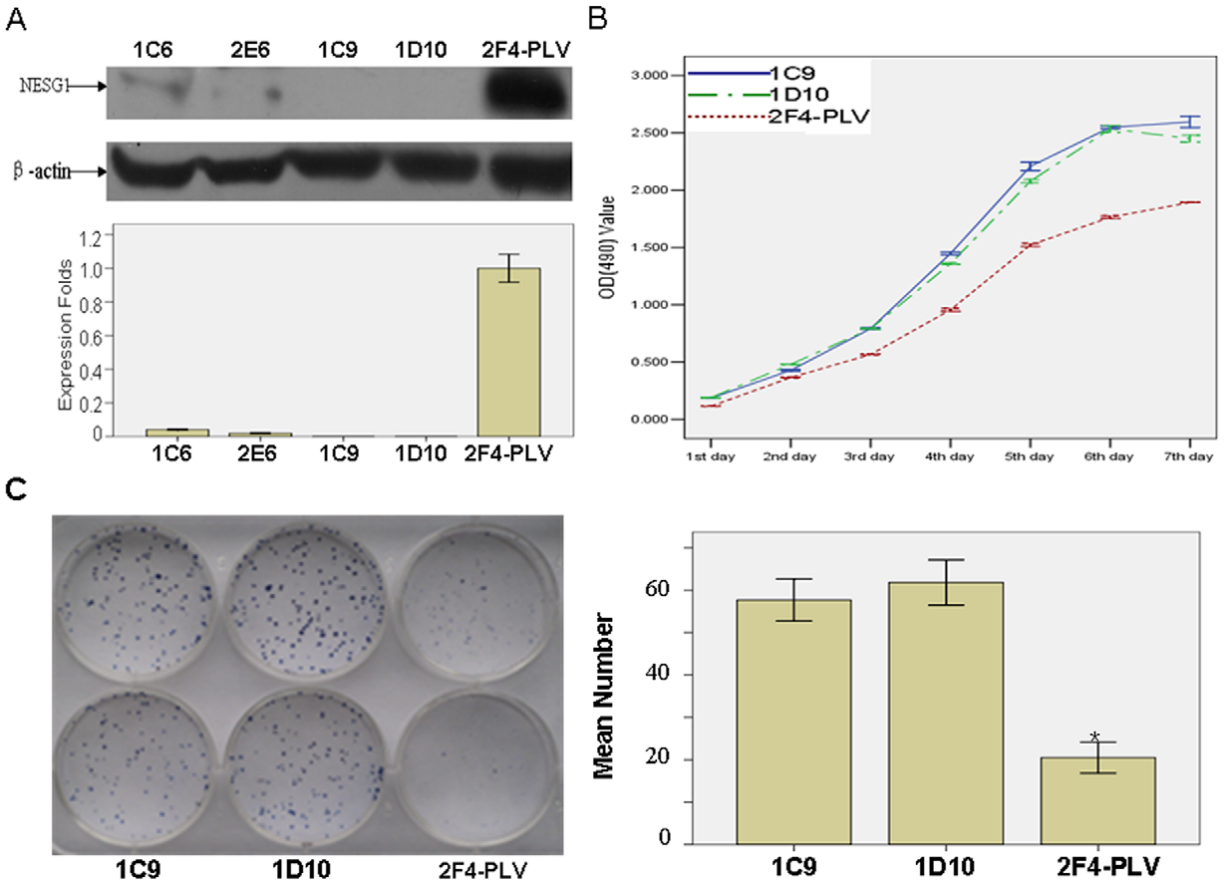
Suppression of NESG1 restores growth and clone formation of NPC5-8F-2F4 cells. **A.** Expression of NESG1 was suppressed in 1C6, 2E6, 1C9 and 1D10 clones compared to 2F4-PLV cells by western blot. **B.** *In vitro* proliferative ability of NPC cells was significantly restored in NESG1-suppressed 1C9 and 1D10 clones compared to NESG1-overexpressing 2F4-PLV cells by MTT assay. **C.** Downregulation of NESG1 markedly increased the clone formation. Data are presented as mean±SD for three independent experiments. (*P<0.05).

To further confirm the biological function of NESG1, we used a lentiviral vector containing shRNA to specifically target and stably knock down NESG1 overexpressed in 2F4 NPC cells. Four stably transfected cell clones were obtained (1C6, 2E6, 1C9, 1D10). Decreased expression of NESG1 protein was confirmed by western blotting in these 4 cells compared to those infected with PLV-Ctr (Figure 3A). The 1C9 and 1D10 cells with significantly reduced NESG1 protein expression were then chosen for further experimentation.

We first examined the effect of decreased NESG1 expression on NPC cell growth *in vitro*. Using MTT assays, we found that shRNA-NESG1 infected 1C9 and 1D10 NPC cells had an elevated growth rate to 2F4-PLV control cells over a seven day period (*P*<0.001)(Figure 3B). These results were also consistent with clonogenicity tests as 1C9 and 1D10 cells formed an increased number of colonies (57.667±4.967 and 61.833±5.345) compared to 2F4-PLV cells (20.500±3.619) over a two-week period. This suggested that NESG1 knockdown dramatically increased colony formation in 1C9 and 1D10 cells (P<0.001)(Figure 3C).

### Knockdown of NESG1 promotes cell migration and invasion

**Figure 4:**
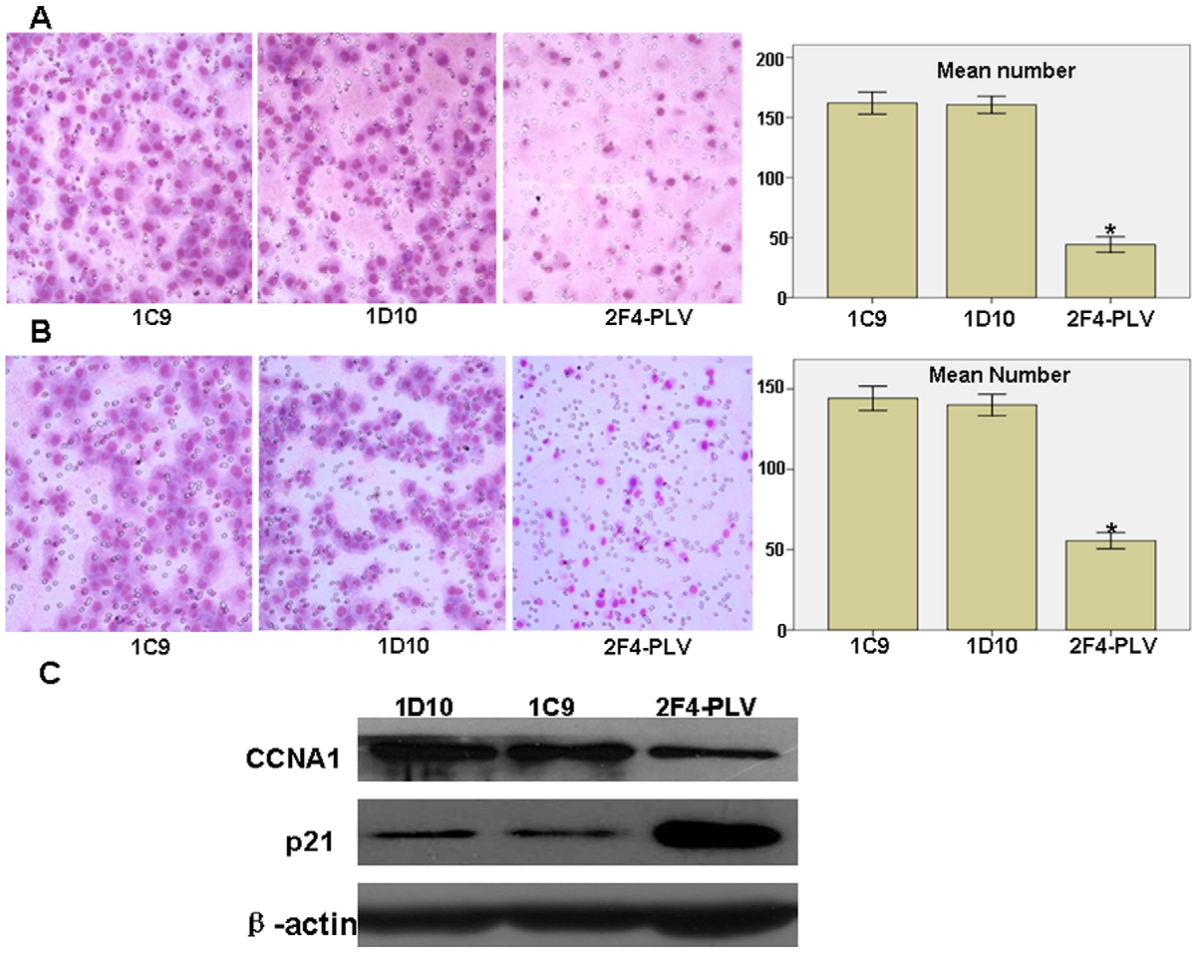
Reduced NESG1 expression promotes cell migration and invasion *in vitro*. **A.** Downregulated NESG1 dramatically enhanced the ability of 1C9 and 1D10 cell migration *in vitro*. **B.** Downregulated NESG1 markedly elevated *in vitro* invasiveness of 1C9 and 1D10 cells. **C.** Suppressing NESG1 expression significantly increased the endogenous protein expression of CCNA1 and reduced the expression of p21 in 1C9 and 1D10 cells. Data were presented as mean±SD for three independent experiments. (*P<0.05).

To further examine the effect of NESG1 on cell migration, 1C9 and 1D10 cells were cultured on a transwell apparatus. After 12-h incubation, the percentage of migrated cells was significantly increased in both 1C9 and 1D10 cell groups compared to the NESG1-overexpressing 2F4-PLV cells (for both P<0.001) (Figure 4A). We next analyzed cell invasiveness using a Boyden chamber coated with Matrigel for 16-h incubation. Both 1C9 and 1D10 cells exhibited significantly increased invasiveness compared to control cells (for both P<0.001)(Figure 4B).

### Suppression of NESG1 expression downregulates p21 and restores CCNA1 levels

In our previous investigation, overexpression of NESG1 promoted the expression of tumor suppressor p21 while downregulating cell cycle promoter CCNA1. We continued examination of these two factors after knockdown of NESG1 in 1C9 and 1D10 NPC cells. Consistent with our prior results, expression of p21 was downregulated while CCNA1 upregulated in 1C9 and 1D10 cells compared to 2F4-PLV cells (Figure 4C).

### NESG1-mediated pathways

**Figure 5:**
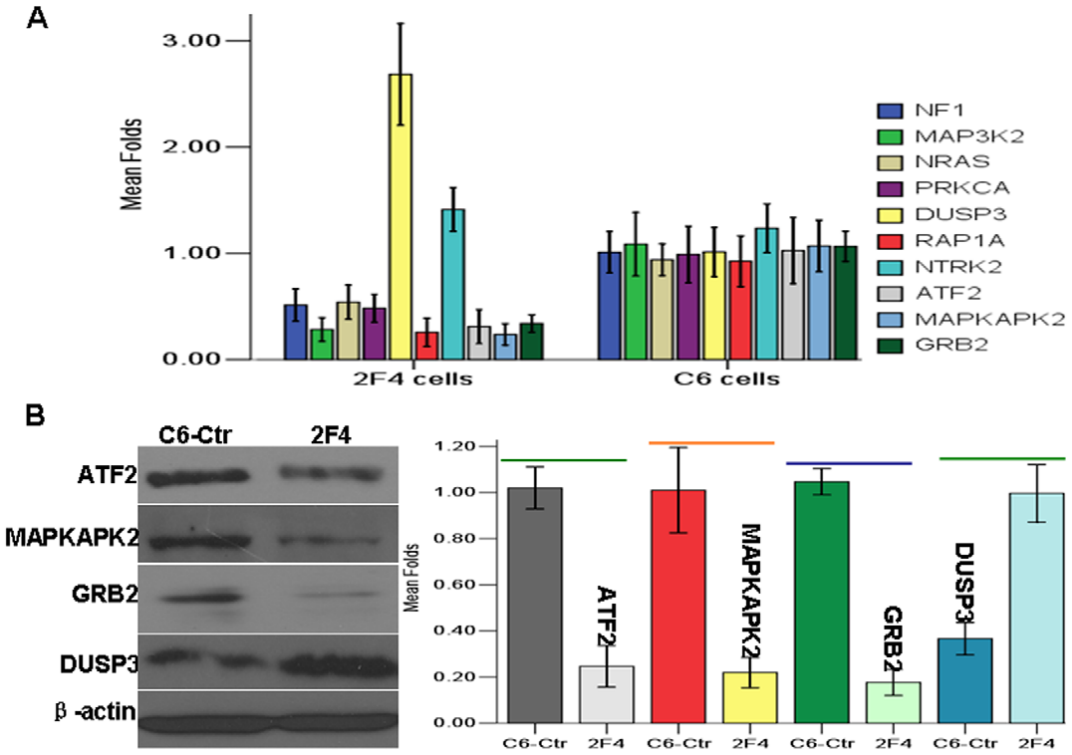
Overexpressed NESG1 regulate the gene expression of MAPK pathway in NPC cells. **A.** Using real-time PCR to validate the differential gene expression of MAPK pathway, DUSP3 (≥2 Fold), NF1, MAP3K2, PRKCA, NRAS, RAP1A, ATF2, MAPKAPK2, and GRB2 (≤0.57 Fold) in NPC 2F4 cells relative to control NPC C6 cells. **B.** Protein expression of ATF2, MAPKAPK2, DUSP3, and GRB2 were consistent with differential expression of mRNA level in NESG1-overexpressing NPC 2F4 cells compared to control NPC C6 cells.

We recently observed 2408 differentially expressed genes between NESG1-overexpressing 2F4 cells and NESG1-negative Ctr-C6 cells (http://www.ncbi.nlm.nih.gov/geo/query/acc.cgi?acc=GSE27318) by microarray analysis [8]. Further, NESG1-mediated differential expression of 1442 genes were used to conduct pathway analysis against the KEGG database, each with an “_at” extension in probe set IDs representing unique probe set sequences. Significant pathways 1–10 are listed in Figure 5A (Table 4), which included cell cycle regulators that had been partially validated [8]. Among them, MAPK signaling was considered to be the most significant pathway mediated by NESG1, especially given its role in cell proliferation, cell migration, and invasion.

**Table 4:** The top 10 pathways mediated by NESG1 in NPC.

Pathway	Number of genes	p-value	Genes involving pathway
MAPK signaling pathway	29	1.14E-15	FGFR2;MAP3K14;MAP3K3;ZAK;MAP2K2;NTF4;PLA2G10;NTRK2;PPM1A;RPS6KA2;DUSP3;DUSP9;ZAK;CRK;MAP4K4;PTPRR;SOS1;NRAS;PRKCA;PPM1B;NF1;FGFR1;FOS;ATF2;MAP3K2;GRB2;ZAK;MAPKAPK2;RAPGEF2;MAX;ZAK;ZAK;RAP1A
Systemic lupus erythematosus	20	1.73E-13	HIST1H4A;HIST1H4B;HIST1H4C;HIST1H4D;HIST1H4E;HIST1H4F;HIST1H4H;HIST1H4I;HIST1H4J;HIST1H4K;HIST1H4L;HIST2H4B;HIST2H4A;HIST4H4;HLA-DMB;TROVE2;H3F3B;LOC644914;H3F3A;HIST1H2BC
Insulin signaling pathway	15	5.91E-09	PRKAA2;PRKAB1;MAP2K2;PIK3R5;CRK;SOS1;NRAS;PRKCI;PRKAB2;PHKB;PPP1CB;RHOQ;GRB2;PRKAA2;RHOQ;PRKAA2;TRIP10;PRKAR2B
Regulation of actin cytoskeleton	18	1.50E-08	FGFR2;BCAR1;ENAH;MAP2K2;PIK3R5;PAK6;ITGA2;CRK;ABI2;RDX;SOS1;NRAS;ITGB8;FGFR1;PIP5K3;WASF1;DIAPH3;ABI2;PPP1CB
Focal adhesion	17	3.52E-08	BCAR1;THBS1;PIK3R5;PAK6;ITGA2;CRK;CAV2;SOS1;PRKCA;ITGB8;ARHGAP5;COL4A2;COL4A1;PPP1CB;COL4A2;GRB2;COL4A4;RAP1A
Cell cycle	13	5.72E-08	CCNB2;E2F1;RBL2;ANAPC1;CHEK1;CDKN2B;RB1;ATM;LOC651610;MCM6;CDC14B;SMC1A;CCNA1
Wnt signaling pathway	14	1.54E-07	PRICKLE1;LRP5;FZD1;CSNK1E;CTNNBIP1;VANGL1;DKK1;WNT9A;DAAM1;CSNK1A1;VANGL2;PRKCA;NFAT5;WNT5A
Non-small cell lung cancer	9	1.63E-07	MAP2K2;PIK3R5;E2F1;PLCG2;SOS1;NRAS;PRKCA;RB1;GRB2
Renal cell carcinoma	10	1.75E-07	MAP2K2;PIK3R5;PAK6;ARNT2;CRK;SOS1;NRAS;EGLN1;GRB2;RAP1A
Jak-STAT signaling pathway	14	1.97E-07	IL24;EPOR;IFNE;PIK3R5;SOS1;LEPR;STAM2;IRF9;GRB2;IL20RB;JAK3;JAK1;IL4R;JAK3;IL2RG

### Expression validation of significant factors associated with MAPK pathway

Using real-time PCR analysis, we examined the expression of 10 genes associated with NESG1-mediated MAPK pathway between 2F4 cells and C6-Ctr cells. With the exception of NTRK2, the other 9 genes including NF1, MAP3K2, PRKCA, NRAS, DUSP3, RAP1A, ATF2, MAPKAPK2, and GRB2 (≥2 Fold or ≤0.57 Fold) were closely matched the expression patterns from the microarray results (Figure 5A) (Table 2). In addition, the protein expression of four of these genes was further validated by western blot. Consistent with the microarray data, ATF2, MAPKAPK2, and GRB2 were markedly downregulated while DUSP3 upregulated in NESG1-overexpressing 2F4 cells compared to NESG1-negative Ctr-C6 cells (Figure 5B).

### No methylation of NESG1 promoter was observed in NPC

**Figure 6:**
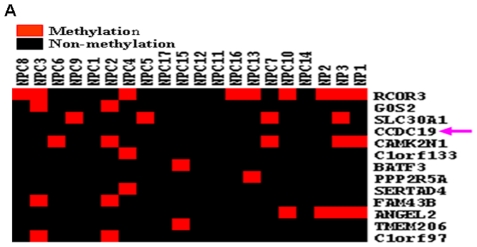
Methylation of NESG1 promoter not observed in NPC samples. **A.** Using a NimbleGen DNA methylation microarray containing NESG1, we did not detect a significant methylation change in NESG1 promoter region in 17 NPC samples and 3 NPs.

Due to a bioinformatics predicted CpG island in the NESG1 promoter (Figure S1), we conjectured that hypermethylation of NESG1 might result in the suppressed expression of NESG1 in NPC. We did not find any methylation modification in NESG1 promoter region in 17 NPC samples or 3 NPs using NimbleGen DNA methylation microarrays (Figure 6A), which suggested that reduced expression of NESG1 in NPC was not due to its promoter methylation.

## Discussion

Nasopharyngeal epithelium-specific gene NESG1 was initially isolated from human normal nasopharynx mucosa using an improved differential display approach, and its sequences was submitted to the Genbank database by our research group in 1999 [8]. In a recent investigation, we updated the NESG1 ORF sequence and studied its role in NPC cells. Our results preliminarily suggested NESG1 function as a tumor suppressor in NPC [9].

In the present study, we presented additional support for this notion as NESG1 protein was downregulated in human NPC tissues and cells compared to noncancerous nasopharynx tissues by western blot. Furthermore, we also found that protein expression of NESG1 was progressively decreased in atypical hyperplasia and cancer tissues compared to normal and squamous epithelium by immunohistochemistry. These results were not only consistent with our previous investigation [9], but also hinted that lost expression of NESG1 was involved in the stages of initiation and precancerous lesion of NPC.

We previously observed that reduced NESG1 protein levels were inversely associated with lymph node metastasis and clinical stage of NPC which suggested its downregulation favored the development of NPC. However, due to the limited patient sample size and the absence of clinical prognosis information, we did not investigate the detailed correlation of NESG1 expression with clinical features and prognosis of NPC. In this report, we used a larger cohort of 204 patients with clinical prognosis information to analyze this relationship. Similar to our previous results, we found that decreased expression of NESG1 inversely correlated with lymph node metastasis and clinical stage of NPC [9]. We further observed that decreased NESG1 expression was correlated with distant metastases and statistically lower in the M1 group compared to the M0 group. Together these studies suggest downregulated NESG1 levels play an unfavorable role in NPC pathogenesis, a correlation which has not been previously reported.

Our investigation provides data that reduced NESG1 protein expression is correlated with decreased NPC patient overall survival. According to univariate analyses, overall survival was significantly correlated with age, TNM classification, and NESG1 expression. Multivariate analyses showed that decreased expression of NESG1 alone could be a significant predictor of poor prognosis for NPC patients. Our data are the first to report the clinical significance of decreased NESG1 expression as an unfavorable prognosis biomarker in NPC.

In a prior study, NESG1 overexpression had been observed to suppress cell proliferation, migration, invasion, and cell cycle progression [9], thus we further examined the biological functions of NESG1 in NPC. We used a loss-of-function approach to knock down the overexpressed NESG1 in 2F4 NPC cells, and confirmed its role in promoting cell proliferation, migration, and invasion *in vitro*. These results are consistent with our previous investigation [9]. Our studies strongly suggest a suppressive role of NESG1 in the development of NPC.

To fully understand the molecular mechanism of NESG1-mediated suppressive pathways in NPC, we analyzed differential expression of NESG1-regulated genes against the pathway-collected database KEGG. Our computational pathway analysis of 1442 differentially expressed genes strongly supported that multiple biological signaling pathways were involved in NESG1-mediated NPC oncogenesis including MAPK, insulin, actin cytoskeleton, focal adhesion, and cell cycle progression. In our recent report, NESG1 altered expression of cell cycle regulators CCNA1 and p21, a finding we confirmed through an inverse approach. We found that inhibition of NESG1 could markedly restore expression of cell cycle promoting CCNA1 while downregulating tumor suppressor p21.

The mitogen-activated protein kinase (MAPK) cascade is a highly conserved module that is involved in various cellular functions, including cell proliferation [10]–[11], differentiation [12] migration [13], and invasion [14], [15]. MAPK was considered to be an important pathway regulated by NESG1 based on its ranking after computer analysis. Real-time PCR was used to validate the differential expression patterns of MAPK pathway-associated genes from the microarray results. Expression of NF1, MAP3K2, PRKCA, NRAS, DUSP3, RAP1A, ATF2, MAPKAPK2, and GRB2 were consistent with the microarray, which suggested that NESG1 participated in the regulation of MAPK pathway in NPC. Four of these relevant genes including ATF2 [16], MAPKAPK2, DUSP3, and GRB2 were selected for further analysis. ATF2 encodes a transcription factor that is a member of the leucine zipper family of DNA-binding proteins. In response to stress stimuli, it activates a variety of gene targets including cyclin A, cyclin D and c-jun, which participate in the oncogenesis of various tissue types. ATF2 expression has been correlated with maintenance of a cancer cell phenotype [17], [18]. MAPKAPK2 is regulated through direct phosphorylation by p38 MAP kinase. In conjunction with p38 MAP kinase, this kinase is known to be involved in many cellular processes including stress and inflammatory response [19], cell cycle regulation [20] and proliferation [21]. A recent report indicated that MAPKAPK2 could mediate p38 mitogen-activated protein kinase (MAPK) activation to drive invasion of bladder cancer by inducing the expression of MMP-2 and MMP-9 [22]. The protein encoded by DUSP3 is a member of the dual specificity protein phosphatase subfamily, which are associated with cellular proliferation and differentiation. Upregulated expression and nuclear localization of DUSP3 promotes the pathogenesis of cervix carcinoma [23]. Loss of the expression can cause cell-cycle arrest and senescence, which was dependent on the hyperactivation of the mitogen-activated protein (MAP) kinases Jnk and Erk [24]. This effect was reversed by Jnk and Erk inhibition or knock-down. Grb2 knockdown reduced mitogen-activated protein kinase (MAPK) activity in BCR-ABL-expressing hematopoietic cells [25]. Tyrosine phosphorylation of Grb2 is taken as a critical mechanism by which PRL antagonizes EGF-induced cell proliferation by attenuating the activation of the Ras/mitogen-activated protein kinase (MAPK) pathway [26]. Consistent with our microarray results, ATF2, MAPKAPK2, and GRB2 were markedly downregulated while DUSP3 upregulated in NESG1-overexpressing 2F4 NPC cells compared to NESG1-negative C6-Ctr cells. Our results suggested a novel mechanism where dysregulated NESG1 participates in the regulation of MAPK pathway in nasopharynx carcinogenesis.

The hypermethylation of CpG islands in gene promoters can often lead to transcriptional silencing of genes, including tumor suppressor genes [27]–[30]. Due to the existence of predicted CpG islands in the NESG1 promoter region, we used a NimbleGen DNA methylation microarray to assess its methylation status in 17 NPC cases and 3 NP samples. However, there were no significant changes in NESG1 promoter methylation observed in these samples, suggesting other mechanisms are involved in the repression of NESG1 in NPC.

In summary, our present study provides additional support that NESG1 functions as a tumor suppressor in NPC. Its decreased expression level as an unfavorable important prognostic factor was negatively correlated with the malignant status of NPC patients. Our findings not only further support that NESG1 regulates cell cycle factors, but also implicate its involvement in the MAPK pathway during NPC oncogenesis. Increased promoter methylation can not account decreased NESG1 expression in NPC, thus a different mechanism is likely responsible for its loss.

## Materials and Methods

### Cell culture, tissue collection and Ethics Statement

Eight (8) NPC cell lines including 5-8F, 6-10B, CNE2, CNE1, C666-1, HONE1, HNE1, and SUNE1 [7] [9] were maintained in RPMI 1640 supplemented with 10% newborn calf serum (NBCS) (PAA Laboratories, Inc, Austria). Three (3) freshly isolated primary NPC tissues, 5 freshly isolated non-cancerous nasopharynx tissues, 204 paraffin-embedded undifferentiated NPC specimens and 149 paraffin-embedded non-cancerous nasopharynx specimens (including 74 normal epithelium, 40 squamous epithelium, and 35 atypical hyperplasia epithelium) were obtained at the time of diagnosis before any therapy from People's Hospital in Zhongshan City, Guangdong, China. In the 204 NPC cases, there were 116 male and 48 female with age ranging from 17 to 76 years (median, 48.98 years). The clinical follow-up time of patients ranged from 4 to 126 months. For the use of these clinical materials for research purposes, prior written informed consent from all the patients (One patient aged 17 also gave consent for himself) and approval from the Ethics Committees of People's Hospital of Zhongshan City were obtained. All specimens had confirmed pathological diagnosis and were staged according to the 1997 NPC staging system of the WHO.

### Western blot

Nasopharynx or NPC tissues were ground in liquid nitrogen and lysed in RIPA buffer on ice containing protease inhibitors. Cells were directly lysed in RIPA buffer. Protein lysates were resolved on 10% SDS polyacrylamide gel, electro-transferred to polyvinylidene fluoride membranes (Invitrogen, Carlsbad, CA, USA), and blocked with 5% nonfat dry milk in Tris-buffered saline, pH 7.5. Membranes were immunoblotted overnight at 4°C with anti-NESG1 polyclonal antibody (Proteintech Group, Inc. Chicago, IL, USA) or p21, anti-β-actin antibody (Santa Cruz Biotechnology, Santa Cruz, CA, USA) or CCNA1, ATF2, MAPKAPK2, GRB2, DUSP3 (Abcam, Landon), followed by their respective HRP-conjugated secondary antibodies. Signals were detected using enhanced chemiluminescence reagents (Pierce, Rockford, IL, USA).

### Immunohistochemistry

Examination of NESG1 expression in samples of undifferentiated NPC and nasopharynx tissues by IHC was performed as previously described [9]. The stained tissue sections were reviewed and scored independently by two pathologists blinded to the clinical parameters. The staining score standard has also been described [9]. For statistical analysis of NESG1 expression in noncancerous tissues against NPC tissues, the staining score of 0, 1∼2, 3∼4, and 5∼6 were respectively considered to be negative, low, medium, and strong expression. For the correlated statistical analysis of NESG1 expression with clinical features and prognosis of NPC patients, the staining score of 0∼4 and 5∼6 were respectively considered to be low and high expression.

### Establishment of stable knock down clones with shRNA-NESG1 from NESG1-expressing NPC 2F4 cell line

Two sequences were selected for targeting the NESG1 gene using the BLOCK-It RNAi Designer (Invitrogen, Carlsbad, CA): NESG1 509 (Sense: 5′-CGCGTCCCCGCGGCAGAAATCCATTCAAAGTTCAAGAGACTTTGAATGGATTTCTGCCGCTTTTTGGAAAT-3′ and Antisense: 5′-CGATTTCCAAAAAGCGGCAGAAATCCATTCAAAGTCTCTTGAACTTTGAATGGATTTCTGCCGCGGGGA-3′) and NESG1 1622 (Sense: 5′-CGCGTCCCCGCATTGAAGCTGAGCGCAAAGTTCAAGAGACTTTGCGCTCAGCTTCAATGCTTTTTGGAAAT-3′ and Antisense: 5′-CGATTTCCAAAAAGCATTGAAGCTGAGCGCAAAGTCTCTTGAACTTTGCGCTCAGCTTCAATGCGGGGA-3′). The preparation of lentiviral vectors expressing human NESG1 short hairpin RNA (shRNA) was performed using the pLVTHM-GFP Lentiviral RNAi Expression System [31] supplied by Prof. Guangfei Xiao who worked in our cancer institute. Replication-incompetent lentivirus was produced by cotransfection of the pLVTHM/NESG1-shRNA expression vector and ViraPower packaging mix containing an optimized mixture of two packaging plasmids, psPAX2 and pMD2G, into 293FT cells. NPC NESG1-overexpressing 2F4 cells were infected with lentiviral particles containing specific or negative control vectors and the single colony with strong GFP expression was selected to establish stable silencing cell lines. The total RNA of these cell clones was isolated, and the levels of NESG1 protein were measured using western blot assays.

### MTT assay

The rate of *in vitro* cell proliferation was assessed using 3-(4, 5-dimethylthiazol-2-yl)-2, 5-diphenyltetrazolium bromide (MTT) assay. Cells were seeded in 96-well plates at a density of 1,000 cells/well. The cells were incubated for 1, 2, 3, 4, 5, 6, or 7 d. Twenty microliters of MTT (5 mg/ml)(Sigma, St. Louis, MO) was added to each well and incubated for 4 h. At the end of incubation, the supernatants were removed, and 150 µl of DMSO (Sigma, St. Louis, MO) was added to each well. The absorbance value (OD) of each well was measured at 490 nm. For each experimental condition, 8 wells were used. Experiments were performed three times.

### Colony formation assay

Cells were plated in 6-well culture plates at 100 cells/well. Each cell group had 2 wells. After incubation for 9 days at 37°C, cells were washed twice with PBS and stained with the Giemsa solution. The number of colonies containing ≥50 cells was counted under a microscope. The colony formation efficiency was calculated as (number of colonies/number of cells inoculated)×100%.

### Cell migration and invasion assays

For the cell migration assay, 1×10^5^ cells in 100 µl RPMI 1640 medium without NBCS were seeded on a fibronectin-coated polycarbonate membrane insert in a transwell apparatus (Costar, MA). In the lower chamber, 600 µl RPMI 1640 with 10% NBCS was added as chemoattractant. After the cells were incubated for 12 h at 37°C in a 5% CO_2_ atmosphere, the insert was washed with PBS, and cells on the top surface of the insert were removed with a cotton swab. Cells adhering to the lower surface were fixed with methanol, stained with Giemsa solution, and counted under a microscope in five pre-determined fields (×200). All assays were independently repeated at least three times. For the cell invasion assay, the procedure was similar to the cell migration assay, except that the transwell membranes were pre-coated with 24 µg/µl Matrigel (R&D Systems, USA) and the cells were incubated for 16 hours at 37°C in a 5% CO_2_ atmosphere. Cells adhering to the lower surface were counted the same way as for the cell migration assay.

### NESG1-mediated pathways in NPC by analyzing gene expression profile data between NESG1-overexpressed 2F4 cells and NESG1-negative Ctr-C6 cells

Gene expression profiles were determined using Affymetrix Human Genome U133 Plus 2.0 Array containing 47,000 transcripts (Affymetrix, Santa Clara, CA) between NESG1-overexpressed 2F4 cells and control Ctr-C6 cells using significance analysis of microarray (SAM) as described previously [9]. NESG1-mediated pathways were analyzed against KEGG database by MAS2.0 software (CapitalBio, Inc, Beijing, China).

### Real-time PCR

The mRNA levels of MAPK signaling regulators NF1, MAP3K2, PRKCA, NRAS, DUSP3, RAP1A, NTRK2, ATF2, MAPKAPK2, and GRB2 in C6-Ctr and 2F4 cells were measured using real-time PCR. ARF5, an invariant housekeeping gene in nasopharynx and NPC samples, was used as internal control [9]. The primer pair sequences for these genes (Table S1) designed spanned at least an intron to distinguish possibly amplified products from genomic DNA. The PCR reaction was carried out using SYBR Green Mix reagent (Takara Inc, Japan). Real-time PCR was repeated three times.

### Examination of NESG1 promoter methylation by DNA methylation microarray assay

The examination procedure of NimbleGen DNA methylation microarray has been described [32]. Briefly, genomic DNA of 17 biopsy NPC samples containing more than 75% of tumor cells and 3 noncancerous nasopharynx epithelium tissues (NPs) was extracted using DNeasy Blood & Tissue Kit (Qiagen, Inc.). Subsequently, all DNA samples were used to perform MeDIP assay as described. MeDIP DNA was utilized to hybridize to NimbleGen C4226-00-01 promoter-tiling arrays that were designed based on the HG18 genome release. All experiments were performed at the Kangchen Biology Corporation, Shanghai, China.

### Statistical analysis

All data were analyzed for statistical significance using SPSS 13.0 software. The Kruskal Wallis Test was used to examine the differences of NESG1 expression among squamous epithelium, normal epithelium, atypical hyperplasia, and cancer tissues of nasopharynx. Mann-Whitney U test was employed to analyze the differences of NESG1 expression between two groups in squamous epithelium, normal epithelium, atypical hyperplasia, and cancer tissues of nasopharynx. Chi-square test was applied to analyze the relationship between NESG1 expression levels and clinicopathologic characteristics. Survival analysis was performed using Kaplan-Meier method. Multivariate Cox proportional hazards method was used for analyzing the relationship between the variables and patients' survival time. One-way ANOVA was used to determine the differences between groups for all *in vitro* analyses. A *P* value of less than 0.05 was considered statistically significant.

## Supporting Information

Figure S1 **Prediction of CpG island in NESG1 promoter.** **A.** CpG island of NESG1 was predicted by methyl primer express software1.0 in promoter sequence of NESG1 containing the first exon 21 bp and its upstream 5048 bp. The results showed a CpG island locating the first exon and its upstream 513 bp.
(TIF)

Table S1 **Differential expression folds of MAPK pathway genes between NESG1-overexpressed NPC 2F4 cells and NESG1-negative NPC C6 cells and their primer pair sequences.**
(DOC)
