# Expression of Concern: Potential Tumor Suppressor NESG1 as an Unfavorable Prognosis Factor in Nasopharyngeal Carcinoma

**DOI:** 10.1371/journal.pone.0231298

**Published:** 2020-04-13

**Authors:** The PLOS ONE Editors

After publication of this article [1], concerns were raised about the identity of the cell lines used in part of the study, the representation of the western blot data, and the availability of the underlying data.

Four of the eight nasopharyngeal carcinoma cell lines used in the western blot experiment shown in Fig 1A have been reported elsewhere to be possibly contaminated or misidentified (CNE1, CNE2, HONE1, and HNE1, see ICLAC Register of Misidentified Cell Lines (iclac.org/databases/cross-contaminations/) [2], Cellosaurus (https://web.expasy.org/cellosaurus/) [3] and [4–5]). These cell lines were not used in the functional and mechanism studies reported in the article. The corresponding author has provided STR profile reports for the 5-8F cells and the HONE1 cells (S1 File).

**Fig 1:**
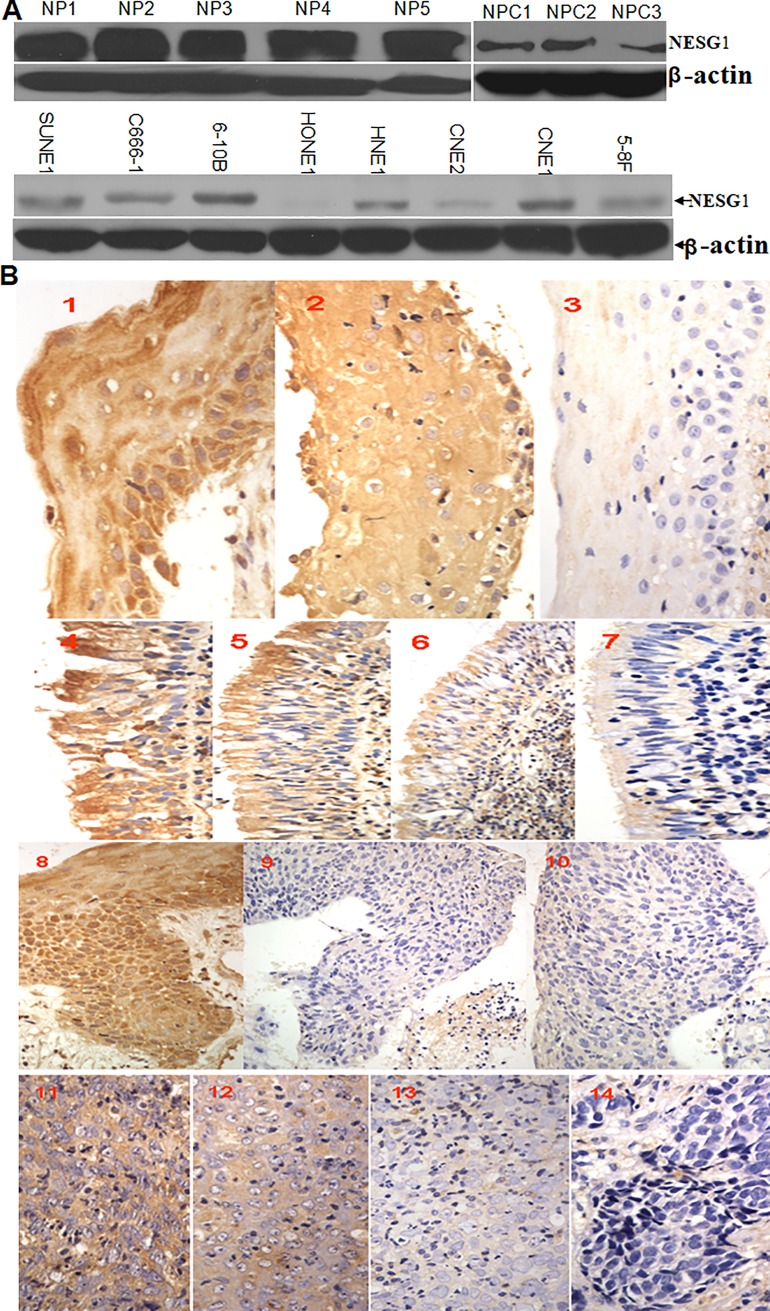
Gradual downregulation of NESG1 protein in normal nasopharynx tissues, atypical hyperplasia, and NPC samples. A. Western blot analysis indicated that NESG1 protein was significantly downexpressed in NPC tissues and NPC cells compared to noncancer nasopharynx epithelial tissues. Exposure time for the film of the NPC samples and the NP samples is about 25 seconds and 20 seconds separately. The actin loading controls were run on the same gels as the target proteins. We cut the blots of target proteins and actin controls separately according to their different molecular weights for further transmembrane and exposure, no stripping or reprobing. B. NESG1 expression was progressively decreased in atypical hyperplasia and NPC samples compared to normal nasopharynx and squamous tissues. **1)** NESG1 expression in squamous epithelium: 1–2: Strong expression, 3: Weak expression; **2)** NESG1 expression in normal epithelium: 4–5: Strong expression, 6: Positive expression, 7: Weak expression; **3)** NESG1 expression in typical hyperplasia tissue: 8: Strong expression, 9–10: Weak expression; **4)** NESG1 expression in NPC: 11: Strong expression, 12: Positive expression, 13: Weak expression, 14: Negative expression.

The results reported in Figs 3–5 in the article use a single cell line (2F4) or its subclones, limiting the generalizability of the findings as representative of nasopharyngeal cancer biology. 2F4 and Ctr-C6 were derived from 5-8F cells [6].

The authors provided clarification of the source and provenance of the cell lines used as follows:

- 5-8F, 6-10B, and SUNE1 are from stock kept at the Cancer Institute of Southern Medical University since 2003, originally obtained from Cancer Center, Sun Yat-sen University where these cell lines were generated [7, 8].
- C666-1 [9] are from stock kept by the Cancer Institute of Southern Medical University since 2003, originally obtained from Cancer Center of Sun Yat-sen University. The C666-1 cell line was originally generated at the Chinese University of Hong Kong.
- CNE1 and CNE2 are from stock kept by the Cancer Institute of Southern Medical University since 1999.
- HONE1 and HNE1 are from stock kept by the Cancer Institute of Southern Medical University, originally obtained from the Cancer Institute of Hunan Medical University (now the Central Southern University) where these cell lines were generated.

In the western blot shown in Fig 1A, there is a vertical discontinuity between lanes 5 and 6. The authors stated that lanes 1–5 and lanes 6–8 originate from different films. A longer exposure time was used for the film for the NPC samples compared to the film for the NP samples. The original uncropped blots underlying Fig 1 are no longer available. A revised Fig 1 is provided here in which the two films are shown on separate panels.

In the NESG1-mediated pathways subsection of the Results, the incorrect reference is included in the in-text citations.

The correct sentences cite Reference 9 of the original article, as follows: “We recently observed 2408 differentially expressed genes between NESG1-overexpressing 2F4 cells and NESG1-negative Ctr-C6 cells (http://www.ncbi.nlm.nih.gov/geo/query/acc.cgi?acc=GSE27318) by microarray analysis [9]. Further, NESG1-mediated differential expression of 1442 genes were used to conduct pathway analysis against the KEGG database, each with an “_at” extension in probe set IDs representing unique probe set sequences. Significant pathways 1–10 are listed in Fig 5A (Table 4), which included cell cycle regulators that had been partially validated [9].”

Reference 9 of the original article is: Liu Z, Li X, He X, Jiang Q, Xie S, et al. (2011) Decreased expression of updated NESG1 in nasopharyngeal carcinoma: Its potential role and preliminarily functional mechanism. Int J Cancer 128: 2562–2571.

The raw data for the NimbleGen DNA methylation microarray experiment reported in Fig 6 were not deposited in a public repository as required by the standards of the field. The dataset was subsequently deposited and is available at: https://doi.org/10.5061/dryad.wh70rxwj1. No additional underlying dataset is available for this article.

The *PLOS ONE* Editors issue an Expression of Concern to alert readers to the concerns about the accuracy of the representation of the western blot data and the use of cell lines previously reported to be misidentified.

## Supporting information

S1 File STR profile reports. STR profiles of HONE1 and 5-8F cell line samples, analyzed in Dec 2017 and Jan 2018, respectively; search results in ATCC and DSMZ databases; and electrophoresis of gene COX1.
(ZIP)

## References

[1] Liu Z, Luo W, Zhou Y, Zhen Y, Yang H, Yu X, et al (2011) Potential Tumor Suppressor NESG1 as an Unfavorable Prognosis Factor in Nasopharyngeal Carcinoma. PLoS ONE 6(11): e27887 10.1371/journal.pone.0027887 22140479 PMC3225374

[2] Capes-Davis A, Theodosopoulos G, Atkin I, Drexler HG, Kohara A, MacLeod RA, et al Check your cultures! A list of cross-contaminated or misidentified cell lines. Int J Cancer. 2010;127(1):1–8. 10.1002/ijc.25242 20143388

[3] Bairoch A. The Cellosaurus, a cell line knowledge resource. J. Biomol. Tech. 2018; 29: 25–38. 10.7171/jbt.18-2902-002 29805321 PMC5945021

[4] Chan SY‐Y, Choy K‐W, Tsao S‐W, Tao Q, Tang T, Chung GT‐Y, et al Authentication of nasopharyngeal carcinoma tumor lines. Int. J. Cancer, 2008; 122: 2169–2171. 10.1002/ijc.23374 18196576

[5] Strong MJ, Baddoo M, Nanbo A, Xu M, Puetter A, Lin Z. Comprehensive High-Throughput RNA Sequencing Analysis Reveals Contamination of Multiple Nasopharyngeal Carcinoma Cell Lines with HeLa Cell Genomes. J Virol. 2014; 88 (18) 10696–10704; 10.1128/JVI.01457-14 24991015 PMC4178894

[6] Liu Z, Li X, He X, Jiang Q, Xie S, Yu X, et al Decreased expression of updated NESG1 in nasopharyngeal carcinoma: Its potential role and preliminarily functional mechanism. Int J Cancer. 2011; 128: 2562–2571 10.1002/ijc.25595 20715168

[7] Song LB, Yan J, Jian SW, Zhang L, Li MZ, Li D, et al Molecular mechanisms of tumorgenesis and metastasis in nasopharyngeal carcinoma cell sublines.Ai Zheng. 2002 2;21(2):158–62. Chinese 12479066

[8] Zhang L, Song L, Ma Y, Huang B, Liang Q, Zeng Y.Differentially expressed gene in nasopharyngeal carcinoma cell lines with various metastatic potentialities. Zhonghua Zhong Liu Za Zhi. 2002 9;24(5):430–4. Chinese 12485491

[9] Cheung ST, Huang DP, Hui AB, Lo KW, Ko CW, Tsang YS, et al Nasopharyngeal carcinoma cell line (C666-1) consistently harbouring Epstein-Barr virus. Int J Cancer. 1999 9 24;83(1):121–6 10.1002/(sici)1097-0215(19990924)83:1<121::aid-ijc21>3.0.co;2-f 10449618

