# Correction to Expression of Concern on: Potential Tumor Suppressor NESG1 as an Unfavorable Prognosis Factor in Nasopharyngeal Carcinoma

**DOI:** 10.1371/journal.pone.0233763

**Published:** 2020-05-20

**Authors:** The PLOS ONE Staff

## Notice of republication

This Expression of Concern [1] was republished on May 8, 2020, to correct errors in the in-text citations for Reference 9 in the seventh paragraph. The in-text citations were linked to the incorrect reference. Additionally, in the second sentence of the ninth paragraph, the link to the NimbleGen DNA methylation microarray dataset was provided as a direct link; in the republished notice this is replaced with a DOI link. The publisher apologizes for these errors. Please download this Expression of Concern again to view the correct version. The originally published notice and the republished, corrected notice are provided here in S1 and S2 Files, respectively, for reference.

## Supporting information

S1 File Originally published, uncorrected Expression of Concern. (PDF)

S2 File Republished, corrected Expression of Concern. (PDF)
