# Supplementary figures and images for: Potential Tumor Suppressor NESG1 as an Unfavorable Prognosis Factor in Nasopharyngeal Carcinoma
Source: PLoS One. 2011 Nov 28;6(11):e27887. doi: 10.1371/journal.pone.0027887 (PMC3225374; doi:10.1371/journal.pone.0027887)

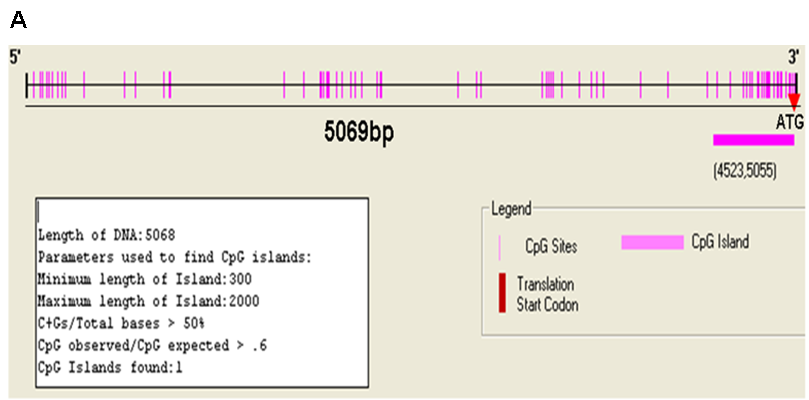

Supplement: Figure S1 — Prediction of CpG island in NESG1 promoter. A. CpG island of NESG1 was predicted by methyl primer express software1.0 in promoter sequence of NESG1 containing the first exon 21 bp and its upstream 5048 bp. The results showed a CpG island locating the first exon and its upstream 513 bp. (TIF) [file pone.0027887.s001.tif]
