# Supplementary material for: Potential Tumor Suppressor NESG1 as an Unfavorable Prognosis Factor in Nasopharyngeal Carcinoma
Source: PLoS One. 2011 Nov 28;6(11):e27887. doi: 10.1371/journal.pone.0027887 (PMC3225374; doi:10.1371/journal.pone.0027887)
Supplement: Table S1 — Differential expression folds of MAPK pathway genes between NESG1-overexpressed NPC 2F4 cells and NESG1-negative NPC C6 cells and their primer pair sequences. (DOC) [file pone.0027887.s002.doc]

| Gene | Differential folds | Primer sequence | Size | Real-time PCR |
| --- | --- | --- | --- | --- |
| NF1 | 0.43 | Sense:5’ TCACATCCTCTGATTGGCAACA3’, Antisense: 5’ TGGCTAACCACCTGGTATAAACAGT 3’ | 196 | 0.51 |
| MAP3K2 | 0.36 | Sense:5’ ACCGTAATGGACATCAGCCC 3’, Antisense: 5’ AGCAGTTTGCCCAATCTCCA 3’ | 71 | 0.26 |
| NRAS | 0.45 | Sense:5’ TTGGGAAAAGCGCACTGACA 3’, Antisense: 5’ TACACAGAGGAAGCCTTCGC 3’ | 203 | 0.57 |
| PRKCA | 0.45 | Sense:5’ TCCGCTCCACACTAAATCCG 3’, Antisense: 5’ ATCAGCTCCGAAACTCCAAAGG 3’ | 157 | 0.49 |
| DUSP3 | 2.10 | Sense:5’ AAGGGCTGCCGACTTCATTG 3’, Antisense: 5’ GGCGATAACTAGCGTTGGGG 3’ | 103 | 2.64 |
| RAP1A | 0.25 | Sense:5’ TGGCATTGAGTAGATCGTCAGTA 3’, Antisense: 5’ TGGCATTGAGTAGATCGTCAGTA 3’ | 232 | 0.28 |
| NTRK2 | 2.3 | Sense:5’ GGAACTGCAGCGAATGACATC 3’, Antisense: 5’ TACATGGCAGCATCAACCAACA 3’ | 296 | 1.38 |
| ATF2 | 0.36 | Sense:5’ CACTCGTTCGACCAGTCACC 3’, Antisense: 5’ ACTGGAGGATGTTGCTGGGT 3’ | 127 | 0.3 |
| MAPKAPK2 | 0.31 | Sense:5’ GCATCCGAATGGGCCAGTATG 3’, Antisense: 5’ AGAATCCTCTGCTCACAACCTG 3’ | 293 | 0.22 |
| GRB2 | 0.35 | Sense:5’ CCACATCCGTTTGGAAACGAT 3’, Antisense: 5’ GGATAAAATCTCCCCGGCGG 3’ | 259 | 0.32 |

**Table S1: Differential expression genes in MAPK pathway and their primer pairs**

Differential folds: Differential expression of microarray between NESG1-overexpressed NPC 2F4 cells and NESG1-negative NPC C6 cells
